# Compensatory Growth of Congenital Solitary Kidneys in Pigs Reflects Increased Nephron Numbers Rather Than Hypertrophy

**DOI:** 10.1371/journal.pone.0049735

**Published:** 2012-11-21

**Authors:** Stefan H. van Vuuren, Chalana M. Sol, Roel Broekhuizen, Marc R. Lilien, Michiel J. S. Oosterveld, Tri Q. Nguyen, Roel Goldschmeding, Tom P. V. M. de Jong

**Affiliations:** 1 Department of Pathology, University Medical Center, Utrecht, The Netherlands; 2 Department of Paediatric Nephrology, University Children’s Hospitals UMC, Utrecht, The Netherlands; 3 Department of Paediatric Nephrology, University Children’s Hospital AMC, Amsterdam, The Netherlands; 4 Department of Paediatric Urology, University Children’s Hospitals UMC Utrecht and AMC, Amsterdam, The Netherlands; University of Tokushima, Japan

## Abstract

**Background:**

Patients with unilateral MultiCystic Kidney Dysplasia (MCKD) or unilateral renal agenesis (URA) have a congenital solitary functioning kidney (CSFK) that is compensatory enlarged. The question whether this enlargement is due to increased nephron numbers and/or to nephron hypertrophy is unresolved. This question is of utmost clinical importance, since hypertrophy is associated with a risk of developing hypertension and proteinuria later in life with consequent development of CKD and cardiovascular disease.

**Methodology/Principal Findings:**

In a cohort of 32,000 slaughter pigs, 7 congenital solitary functioning kidneys and 7 control kidneys were identified and harvested. Cortex volume was measured and with a 3-dimensional stereologic technique the number and volume of glomeruli was determined and compared. The mean total cortex volume was increased by more than 80% and the mean number of glomeruli per kidney was 50% higher in CSFKs than in a single control kidney, equaling 75% of the total nephron number in both kidneys of control subjects. The mean total glomerular volume in the CSFKs was not increased relative to the controls.

**Conclusions/Significance:**

Thus, in pigs, compensatory enlargement of a CSFK is based on increased nephron numbers. Extrapolation of these findings to the human situation suggests that patients with a CSFK might not be at increased risk for developing hyperfiltration-associated renal and cardiovascular disease in later life due to a lower nephron number.

## Introduction

Patients with unilateral MultiCystic Kidney Dysplasia (MCKD) or unilateral renal agenesis (URA) have a congenital solitary functioning kidney (CSFK). Many have observed compensatory enlargement of CSFKs in utero.[1]–[3] This observation raises the question whether the nephrons of a CSFK are necessarily hyperfiltrating and hypertrophic, with the associated risk of developing hypertension and kidney failure at later age, or hyperplastic, with more but normal nephrons. Analysis of a single case of CSFK by Maluf [4] suggested already an increase in nephron number in a CSFK, but, considering the 12.8 fold variation of nephron numbers seen in healthy individuals [5], it is difficult to extrapolate this observation to other CSFK patients.

According to the hyperfiltration hypothesis, a reduced nephron number is associated with hyperfiltration and hypertrophy of the remaining nephrons leading to systemic hypertension, proteinuria and glomerulosclerosis. [6], [7] Indeed, a 50% reduction in the number of nephrons in adults with two kidneys, is a risk factor for the development of hypertension. [8].

A number of studies in human adults with either URA or surgical loss of one kidney in childhood have reported an increased incidence of hypertension even when the remaining kidney was considered “normal”.[9]–[11] However, it should be realized that other risk factors might have confounded these results. In particular, patients with a CSFK and low birth weight are known to have low nephron numbers [12] which makes them prone to developing microalbuminuria due to hyperfiltration. [13] Also patients being overweight and having a CSFK are more prone to develop renal failure and proteinuria, [14] which might be largely weight-dependent, since obesity-related hyperfiltration is known to improve considerably upon weight reduction. [15] Thus, for development of hyperfiltration and associated health risks, nephron number in relation to total body mass appears to be more important than absolute nephron number. Moreover, some of the studies cited had also included patients in whom the dysplastic kidney had not been removed, and might have been the source of systemic hypertension as well as glomerular hyperfiltration in the contralateral kidney.

Nephron development in humans begins in the ninth week of gestation. Proliferation of nephrons is particularly rapid in the last trimester, continuing into the 36^th^ week of gestation [16]. In an ultrasound study we have demonstrated that compensatory enlargement occurs in 88% of the fetuses with a CSFK. This compensatory enlargement is being evident from the 20^th^ week of gestation onward, and results in a mean enlargement of 11.3% in length by the 37^th^ week of gestation [17]. Thus, early unilateral disturbance of kidney development might allow for at least some compensatory increase in nephron number (i.e. hyperplasia) of the CSFK, but it is unclear whether this would be sufficient to eliminate the (patho-) physiological stimulus for glomerular hyperfiltration and nephron hypertrophy.

In this study, widely available pig kidneys, comparable to human kidneys in structure, relative size and function were used as a model for human kidneys. [18].

Because current technology does not allow for accurate determination of nephron size and number in human patients with a CSFK, we set out to explore this issue in a comparative analysis in slaughterhouse pigs with one or two kidneys.

## Methods

### Study Objects

The CSFKs and the control kidneys were obtained by veterinary supervisors responsible for the official meat inspection (H. Rooijakkers and P. van Krieken) from 26 week old pigs that were slaughtered for meat consumption (VION Boxtel B.V.) and were stored on ice for 1–2 days. In a cohort of 32,000 slaughter pigs, 7 were identified to have a congenital monokidney without other anomalies. For comparison 7 kidneys were harvested from random control pigs in the same cohort.

### Number and Volume Estimation

The number and volume of glomeruli were estimated with use of a modified 3-dimensional approach presented by the group of Kerstin Amann. [8] After halving the kidneys, they were immersed and fixated in formaldehyde. After weighing, the entire kidney was cut into 2-mm slices in a cranial-to-caudal direction, and the medullary pyramids were removed from the cortex. The cortex was weighed, and the cortical volume was determined using the volume-replacement method. To avoid bias, fifteen random blocks of 6 by 6 mm throughout the entire cortex were dissected for further analysis from 15 random 2 mm slices. The blocks (of 6 by 6 by 2 mm) were embedded in paraffin and serially sectioned (3 µm) and PAS stained. Shrinkage due to embedding was measured.

Every first and eighth section from each block was selected for analysis. An area touching the left and bottom side of the section plane measuring 4.18 by 3.53 mm (1280 by 1080 pixels) of the selected sections was photographed using a Nikon Eclipse E800 microscope with a Nikon DXM1200 digital camera using the Nikon ACT-1 software version 2.70 (Nikon Netherlands, Lijnden, The Netherlands). The photographs (original magnification 20x) were analyzed full screen on a 22 inch HP Compaq LA2205 WG screen.

Only those glomeruli were counted that appeared in the first plane but not in the eighth plane, and that did not touch the forbidden lines (bottom and right).

To estimate the mean glomerular volume per kidney, we outlined Bowman’s capsule of each individual glomerulus in the first plane using the image analysis software ImageJ (v1.44o Rasband WS, U. S. National Institutes of Health, Bethesda, Maryland U) to determine the total glomerular area found in the section. The sampling volume was calculated by multiplying total tissue area by the thickness of the section (e.g., 3 µm * 8 sections = 24 µm). A correction for tissue shrinkage (29%) was made, and the resulting volume, multiplied by the weight of the fixed kidney, yielded the mass of the portion of the cortex being examined (m_exam_). The weight of the cortex being examined was divided by the weight of the total cortex (M_total_) to calculate the ratio. The number of glomeruli was calculated using the following equation: number = 1 : (m_exam_ : M_total_) * ∑Q-, where Q- is the number of glomeruli found in the first section but not in the eight section.

Nephron volume was calculated by dividing total kidney volume by the number of glomeruli.

The number of papillae of CSFKs was determined by close inspection of the pyelum in a longitudinally halved kidney and compared to the number of papillae of control kidneys. Of these halved kidneys the total area, the cortical area, the pyelum area and the surface area of the medullary pyramids was determined using ImageJ ((v1.44o Rasband WS, U. S. National Institutes of Health, Bethesda, Maryland U).

### Histologic Examination of the Kidney

In addition to the stereologic analysis, all kidneys were examined by light microscopy. The number of heavily sclerosed glomeruli per 100 glomeruli in the cortex was counted.

### Glomerular Cell Number

To estimate the number of cells within the glomeruli, 3 µm thick slides were stained for WT-1 (NCL-L-WT1-562, Leica) to distinguish the podocytes from the other glomerular cells. For each kidney, the number of podocytes and the total cell number were determined in 10 glomerular tuft cross sections. (Count tool, Adobe Photoshop CS5).

### Intra- and Inter- Observer Variation

For determination of intra- and inter-observer variation, two investigators familiar with the 3-D stereologic method, both examined one kidney three times, being unaware of the kidney being CSFK or control. The coefficient of variation was calculated.

### Statistical Analysis

The results are expressed as mean+/− standard deviation. The statistical significance of results was calculated with a student’s t-test.

## Results

### Number and Volume Estimation

**Figure 1:**
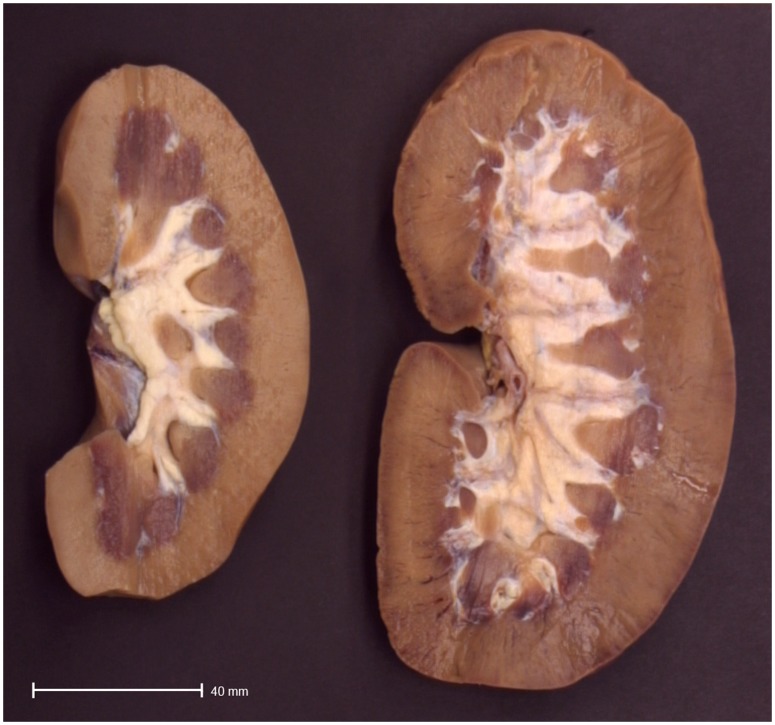
Macroscopic view of a solitary kidney compared to a control kidney. Representative demonstration of size of a normal kidney (left) compared to a solitary kidney (right).

The mean total weight of the CSFKs was 84 percent higher than that of control kidneys (Table 1). The cortex weight and volume were 81 and 83 percent higher in the CSFKs than in the control kidneys (Table 1, Figure 1).

**Table 1:** Characteristics of the kidneys.

Control Kidneys											
	Sex	KidneWeight*	Cortex Weight*	Cortex Volume	Glomeruli #	Mean Glomerular Volume	Total Glomerular Volume	Mean Nephron Volume	Cells per Glomerular Tuft Cross Section	Podocytes Glomerular Tuft Cross Section	Percentage of Podocytes per Glomerular Tuft Cross Section
	*m/f*	*g*	*g*	*ml*	*n*	*x10* ^−3^ *mm* ^3^	*mm* ^3^	*mm* ^3^	*n*	*n*	*%*
Control-1	f	161	129	128	1,226,839	5.58	9,787	0.13	115.6	21.6	18.7
Control-2	m	162	136	136	812,336	5.90	6,850	0.20	138.9	19.1	13.8
Control-3	m	166	140	142	1,735,799	6.35	15,747	0.10	118.3	25.1	21.2
Control-4	m	174	153	155	987,884	6.18	8,728	0.18	166.2	28.3	17.0
Control-5	f	177	152	153	1,827,757	6.67	17,409	0.10	137.0	20.1	14.7
Control-6	m	187	160	162	1,856,511	5.36	9,951	0.10	111.1	18.3	16.5
Control-7	f	193	161	173	2,306,990	6.10	20,113	0.09	112.3	21.7	19.3
***Mean***		***174***	***147***	***149***	***1,536,302***	***6.02***	***12,654***	***0.13***	***128.5***	***22.0***	***17.3***
*+/− SD*		*+/−12,4*	*+/−12,3*	*+/−15,6*	*+/−538,809*	*+/−0.44*	*+/−5,040*	*+/−0.05*	*+/−20.2*	*+/−3.5*	*+/−2.6*
*p-value for comparison*		*<0.001*	*<0.001*	*<0.001*	*0.008*	*0.56*	*0.03*	*0.43*	*0.34*	*0.61*	*0.67*
**Congenital Solitary Functioning Kidneys**											
	**Sex**	**Kidney** **Weight** *	**Cortex** **Weight** *	**Cotex** **Volume**	**Glomeruli**	**Mean Glomerular Volume**	**Total Glomerular Volume**	**Mean** **Nephron** **Volume**	**Cells per Glomerular** **Tuft Cross** **Section**	**Podocytes Glomerular** **Tuft Cross** **Section**	**Percentage of Podocytes per Glomerular Tuft Cross Section**
	***m/f***	***g***	***g***	***ml***	***n***	***x10*** **^−3^** ***mm*** **^3^**	***mm*** **^3^**	***mm*** **^3^**	***n***	***n***	***%***
CSFK-1	f	275	227	227	2,031,045	6.56	19,036	0.16	154.1	28.8	18.7
CSFK-2	f	276	225	231	2,208,398	5.82	18,368	0.13	174.0	31.6	18.2
CSFK-3	m	300	251	266	2,771,222	7.28	28,833	0.11	122.1	23.3	19.1
CSFK-4	m	325	276	283	1,808,309	5.04	13,013	0.18	122.4	16.4	13.4
CSFK-5	m	345	277	282	2,624,535	5.28	19,805	0.13	96.9	17.9	18.5
CSFK-6	m	356	298	303	2,288,602	4.50	14,703	0.16	147.6	26.0	17.6
CSFK-7	m	380	320	323	2,377,973	6.02	20,467	0.16	176.2	19.5	11.1
***Mean***		***322***	***268***	***274***	***2,301,441***	***5.79***	***19,175***	***0.14***	***141.9***	***23.4***	***16.6***
*+/− SD*		*+/−40,5*	*+/−35,5*	*+/−35,4*	*+/−330,670*	*+/−0.95*	*+/−5,066*	*+/−0.02*	*+/−29.4*	*+/−5.7*	*+/−3.1*

* The weight of the fixed tissue is given.

# Per kidney. Comparing two control kidneys with one CSFK by multiplying the demonstrated number found in control kidney by 2 would yield a total glomerular number of 3,072,604+/−1,077,619 for controls and 2,301,441+/−330,670 for CSFKs (p = 0.10).

The mean number of glomeruli counted per field was 4.31+/−2.41. The mean number of glomeruli counted per pig was 64.77+/−19.14. The mean number of glomeruli was 50 percent higher in the CSFKs than in the control kidneys, but the mean glomerular volume was not increased (Table 1). The mean number of glomeruli in the CSFKs was 2,301,441+/−330,670 compared to 1,536,302+/−538,809 in the control kidneys.

**Figure 2:**
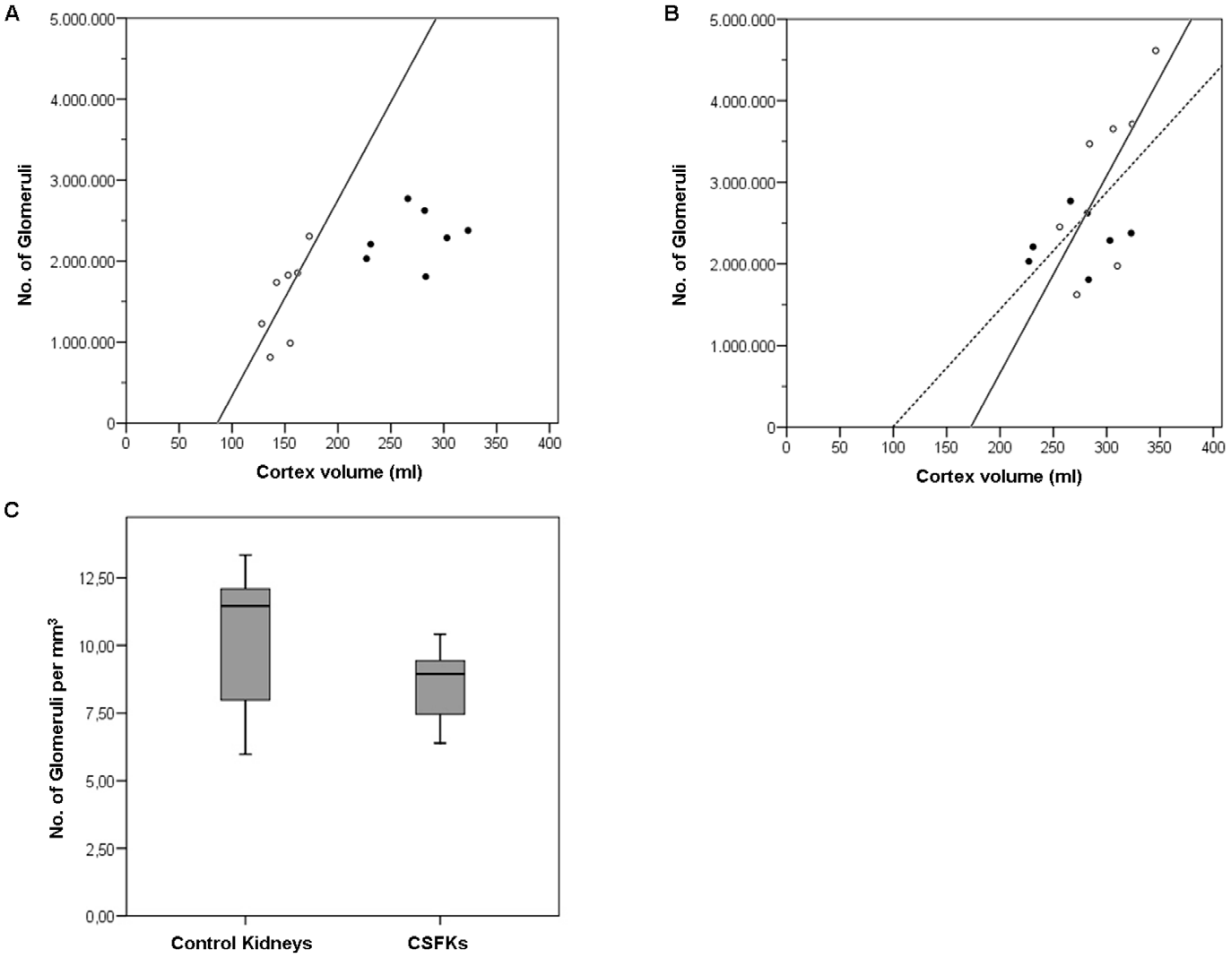
Correlation between number of glomeruli and cortex volume. A. Comparison between number of glomeruli and cortex volume per kidney of congenital solitary functioning kidneys (CSFKs, closed circles) and control kidneys (open circles) at 26 weeks of age with regression line for control kidneys (solid line, R = 0.697, P = 0.082). B. Comparison between number of glomeruli and cortex volume per pig at 26 weeks of age. Pigs with a CSFK are depicted by closed circles, and control pigs by open circles. Values for control pigs are extrapolated from one to two kidneys. Regression lines are shown for control kidneys (solid line, R = 0.697, P = 0.082) and for controls as CSFKs together (dotted line, R = 0.577, P = 0.31). C. Comparison between median number of glomeruli per mm^3^ in CSFKs and control kidneys. Median number of glomeruli per mm^3^ in CSFKs was 8.95 (range 6.39–10.42) and in control kidneys 11.46 (range 5.97–13.34).

As shown in figure 2A, the increase in cortex volume (83%) of CSFKs exceeded their increase in glomerular number (50%). As illustrated in figure 2B, the total cortical volumes and glomerular numbers of CSFKs were, although generally lower, mostly within the range of two controls. In figure 2C, the median of the number of glomeruli per mm^3^ for CSFKs and control kidneys are shown. In CSFKs, the mean number (8.51) of glomeruli per mm^3^ did not differ significantly from the mean number (10.13) of glomeruli per mm^3^ in the control kidneys (p = 0.21).

**Figure 3:**
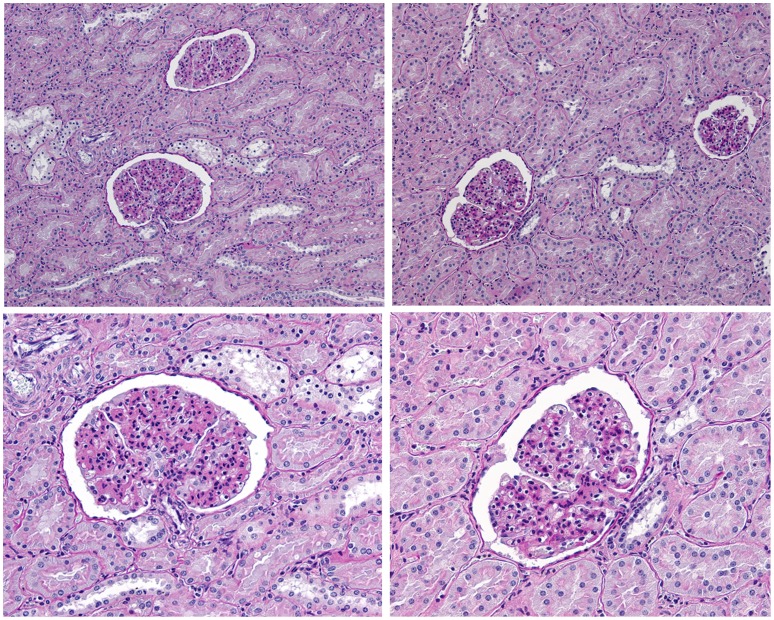
Microanatomy of a solitary kidney compared to a control kidney. Representative pictures of the microanatomy of a normal kidney (left) compared to a solitary kidney (right). The upper pictures demonstrate an overview of the microanatomy of the glomeruli and tubules (original magnification 100x). The lower pictures demonstrate a detailed view of the glomeruli (original magnification 200x).

The higher number of glomeruli in combination with a comparable microanatomy (Figure 3) resulted in a 52 percent higher total glomerular volume in the CSFKs than in the control kidneys (Table 1). The mean total glomerular volume was in the CSFKs 19,175 mm^3^+/−5,066 mm^3^ compared to 12,654 mm^3^+/−5,040 mm^3^ in the control kidneys.

As demonstrated in Table 1, mean volume of individual nephrons in CSFKs 0.14 mm^3^+/−0.02 was comparable to that of control kidneys 0.13 mm^3^+/−0.05.

The number of papillae per kidney in CSFKs (11.4+/−1.52) was increased by 39% compared to control kidneys (8.2+/−0.45). The total surface area in CSFKs was 105.2 cm^2^+/−21.0 compared to 71.6 cm^2^+/−9.3 in control kidneys. The percentage surface area comprised of total of cortex, medullary pyramids, and pyelum was 54.2%+/−4.8; 17.6%+/−3.9; and 27.5%+/−5.4 in CSFKs and 51.9%+/−5.3; 20.6+/−3.3; and 27.5+/−3.0 in control kidneys respectively.

### Histologic Examination of the Kidney

There was no difference (p = 0.76) between the percentage of sclerosed glomeruli in CSFKs (mean 0.57%) and the control kidneys (mean 0.71%).

### Glomerular Cell Number

**Figure 4:**
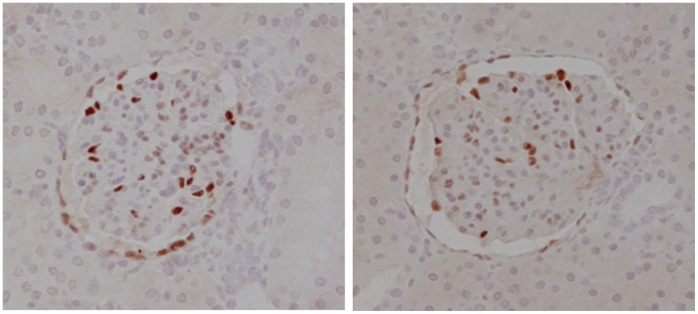
Podocytes in glomerular tuft cross section. Representative pictures WT-1 stained glomerular tuft cross sections of a normal kidney (left) compared to a solitary kidney (right). The pictures demonstrate an overview of the ratio of podocytes (red) versus the number of other glomerular cells (blue, original magnification 200x).

As shown in table 1 and figure 4 the mean number of cells per glomerular tuft cross section in CSFKs (141.9+/−29.4) was comparable to the number found in control kidneys (128.5+/−20.2). Also the number of podocytes per cross section and per total glomerular cell number was comparable in CSFKs and control kidneys (23.4+/−5.7; 16.6%+/−3.1, and 22.0+/−3.5; 17.3%+/−2.6, respectively).

### Intra- and Inter- Observer Variation

Intraobserver coefficients of variation were 1.19 percent and 2.72 percent, respectively, and interobserver coefficient of variation was 3.00 percent.

## Discussion

In this study we found evidence that, in pigs, compensatory enlargement of a CSFK is largely due to an increased number of nephrons, which averaged 150% of the mean number of glomeruli in a single control kidney, or 75% of the mean total number in control subjects. This 75% clearly overlaps with the wide variation (from 210,332 to 2,702,079) in total nephron numbers per kidney found in healthy individuals with two kidneys [5].

The increase in cortex volume (83%) of CSFKs exceeded their increase in glomerular number (50%), which suggests hypertrophy of nephrons. However, the increase of nephron volume by 13% in CSFKs was not significant (p = 0.43), and the most critical parameter, glomerular volume, was increased by only 4%, which was also not significant (p = 0.56). Moreover, the total cortical volumes and glomerular numbers of CSFKs were, although generally lower, mostly within the range of controls. The number of glomeruli per mm^3^ does not differ significantly between CSFKs and control kidneys. The glomerular size, the mean number of cells, podocytes and the percentage of podocytes per glomerular tuft cross section were all comparable in both groups. Based on our observations the increase in nephron volume of the CSFK is due to the wide variation in nephron volume and the majority of the compensatory enlargement is due to nephron hyperplasia.

Remarkably, this increase in kidney weight (84%) and nephron number (50%) in pig CSFKs in our analysis corresponds nicely with previously observed increase in kidney weight (80%) and nephron number (56%) in a single human case of CSFK reported by Maluf. [4].

Douglas-Denton et al. [19] reported on incomplete compensation for nephron loss in male ovine fetuses that underwent unilateral nephrectomy at 100 days of gestation and were sacrificed after 27 to 34 days. They observed a 45 percent increase in the number of nephrons in the kidneys from the unilaterally nephrectomized, as compared to sham operated sheep, leaving a deficit of 27% in total nephron number. Remarkably, and in contrast to our own observations in CSFK pigs, the mean glomerular volume in the unilaterally nephrectomized sheep was 26 percent lower than in controls and total glomerular volume per individual kidney was comparable in both groups. This apparent discrepancy might relate to the fact that in the cited study [19] fetal kidneys were investigated, while we analyzed kidneys from 26 week old pigs, suggesting that compensatory growth (i.e. normalization of glomerular size) is largely due to an increase in nephron number during fetal development while hypertrophic glomerular growth occurs after birth.

Moreover, in a related study, female sheep that underwent fetal unilateral nephrectomy at 100 days of gestation had elevated blood pressure at 6 and 12 months of age as compared to sham operated sheep. [20] From clinical studies it is learned that apparently the compensatory growth is enough to maintain a normal blood pressure during life for most subjects with a CSFK. [21].

In contrast to the increase in glomerular number by 50% in this study and the increase by 45% demonstrated by Douglas-Denton et al. [19], Amakasu et al. demonstrated a more limited, 26% increase of glomerular number in rats. [22] However, both male and female rats evaluated in that study had generally more extensive developmental defects of the URA than most human cases of CSFK and than the pigs we studied. Specifically, several males had undescended testis with agenesis and hypoplasia of the accessory organs, while complete and partial agenesis of the uterine horn was observed in several female rats. [23] In addition to the increase of glomerular number the rats also demonstrated pathological changes associated with progressive renal insufficiency. [22] Although it is a beautifully executed study, the unilateral urogenital anomalies rat strain with multiple congenital defects might not be the best model for patients with a CSFK, especially for those without additional anomalies.

One of the most intriguing observations we made when evaluating the structural morphology was the 1.39 fold higher number of medullary papillae in the CSFKs as compared to normal control kidneys. This suggests an impact of unilateral kidney agenesis or dysplasia on uretric bud arborization at an early stage in the development of the contralateral kidney. Nephron number is known to be influenced by ureteric bud arborization downstream of PAX2 [24], RET [25] and the ALD-H1A2 [26] activity. In addition, a recent study [27] provides evidence that effects of the OSR1 allele are additive with genes that alter ureteric bud branching.

We acknowledge several limitations of this study. First, the glomerular function of the kidneys we studied was not known. Although glomerular function would make the results more appealing, due to the extensive size of the cohort (32,000) and legislation on animal experiments we were unable to perform functional analysis. Second, the pathogenesis of the CSFKs remains doubtful (e.g. URA or MCKD). Future studies including separation of the two groups could provide useful information. Third, the tissue samples were embedded in paraffin, which caused 29% tissue shrinkage. This extensive shrinkage is thought not to interfere with the results obtained because of isometric shrinkage of all structures. Strength of this study is the size of the cohort from which the kidneys were obtained.

In summary, our study demonstrates that the total nephron number of CSFK pigs is comparable to the nephron number of healthy pigs, and mean compensatory hypertrophy and subsequent hyperfiltration is limited. This might raise the question whether it is mandatory to subdue CSFK patients to long term follow up. However, the considerable interindividual difference in nephron number observed in CSFK pigs and human subjects suggests that a yet unknown percentage of human CSFK patients is at risk for developing hyperfiltration and hypertrophy-associated renal and cardiovascular disease. As for now, non-invasive determination of nephron number and size, and detection of hyperfiltration before occurrence of associated renal damage are not yet feasible. Until it is possible to identify which of the patients with a CSFK are at risk long-term follow-up is still indicated.

Since we see a correlation between the kidney volume and the number of nephrons, a good first step would be to monitor the kidney volume of a solitary kidney for compensatory enlargement during pregnancy and compare them to the appropriate charts [28].

In conclusion, the mechanism underlying hyperplasia and hypertrophy in CSFKs remains unknown but might include as yet unknown feedback governing branching morphogenesis in early papillary bud arborization. Elucidation of relevant cellular signalling pathways might help to identify opportunities for future diagnostic and intervention tools to identify subjects at risk and maybe support increase of nephron numbers in CSFKs beyond the danger zone.
